# A Comparative Study of the Acute Toxicities of Three-Dimensional Conformal Radiotherapy (3D-CRT) Versus Intensity-Modulated Radiotherapy (IMRT) in Locally Advanced and Inoperable Oral Cavity Cancer

**DOI:** 10.7759/cureus.115752

**Published:** 2026-09-03

**Authors:** Mohammad Rahamat Ullah, Uday Kumar Sarkar, Syed Md. Asadul Hoque, Newton Kumar Sarker

**Affiliations:** 1 Department of Radiotherapy, Manikganj Medical College Hospital, Dhaka, BGD; 2 Department of Oncology, Chattogram Maa-O-Shishu Hospital Medical College, Chattogram, BGD; 3 Department of Radiotherapy, Dhaka Medical College Hospital, Dhaka, BGD; 4 Department of Radiotherapy, Shaheed Ziaur Rahman Medical College Hospital, Bogura, BGD

**Keywords:** 3d-crt, dysphagia, imrt, locally advanced and inoperable oral cavity cancer, xerostomia

## Abstract

Background

Oral cavity cancer is a substantial and increasing public health burden in Bangladesh due to widespread tobacco abuse and betel nut chewing. According to GLOBOCAN 2022, oral cavity cancer is the second most common cancer in Bangladesh after esophageal cancer. Locally advanced and medically inoperable oral cavity cancer can be treated with concurrent chemoradiotherapy (CCRT) as an established standard of care, which can be delivered using either three-dimensional conformal radiotherapy (3D-CRT) or intensity-modulated radiotherapy (IMRT) techniques.

Methods

This quasi-experimental study was carried out among 84 patients with locally advanced and inoperable oral cavity cancer (LAIOCC) from December 2022 to October 2023 who fulfilled the eligibility criteria and were equally distributed (42 in each arm) into two treatment arms. Arm A received CCRT with 3D-CRT and weekly cisplatin (40 mg/m²), whereas Arm B received IMRT with weekly cisplatin (40 mg/m²). Follow-up was conducted weekly during chemoradiotherapy and at the sixth week after completion of treatment.

Results

Xerostomia was observed more frequently in the 3D-CRT arm than in the IMRT arm (p = 0.04). Similarly, dysphagia occurred more often in the 3D-CRT arm than in the IMRT arm (p = 0.02). However, the difference in mucositis was not statistically significant (p = 0.22). Likewise, the difference in dermatitis between the two arms was not statistically significant (p = 0.63). Acute hematological toxicities, including anemia (p = 0.75), leukopenia (p = 1.00), and thrombocytopenia (p = 0.52), also did not differ significantly between the arms.

Conclusion

IMRT was associated with a lower incidence of dysphagia and xerostomia than 3D-CRT. Therefore, the IMRT technique provides a significant benefit to patients with oral cavity carcinoma compared with the 3D-CRT technique in terms of acute toxicities, particularly xerostomia and dysphagia.

## Introduction

Head and neck cancers (HNCs) comprise a diverse group of malignancies that originate from the upper aerodigestive tract, including the lips, oral cavity, nasal cavity, paranasal sinuses, pharynx, larynx, and salivary glands [1]. Oral cavity cancer includes cancers of the upper and lower lip, gingivobuccal sulcus, buccal mucosa, upper and lower gingiva, retromolar trigone, hard palate, floor of the mouth, and anterior two-thirds of the tongue. According to global cancer statistics from 2020, cancers of the lip, oral cavity, and pharynx accounted for approximately 4% of all new cancer cases worldwide [2]. In Bangladesh, data from GLOBOCAN 2022 revealed that lip and oral cavity cancers ranked as the second most prevalent cancer type, following esophageal cancer, with 16,083 newly diagnosed cases, representing 9.6% of the national cancer burden [3]. Important risk factors for the development of oral cavity cancer in Bangladesh and other South Asian countries include the use of tobacco, alcohol, poor oral hygiene, and betel quid chewing [4].

Surgical resection, radiotherapy (RT), chemotherapy, or combined-modality approaches are classical treatment options that have been employed for patients with cancer of the oral cavity. The choice of treatment modality, either singly or in combination, depends on the stage, size, and extension of the primary tumor and relevant patient factors such as age, performance status, and comorbidities. According to a study evaluating National Comprehensive Cancer Network (NCCN) guidelines, surgery is recommended for early-stage and locally advanced resectable lesions in oral cavity cancer. After surgery, postsurgical adjuvant RT as a form of concurrent chemoradiotherapy (CCRT) or RT alone is required if adverse features are present, including extranodal extension (ENE) and/or a pathologically positive margin, close margin, pT3-4 tumors, pN+, lymphovascular invasion, or perineural invasion [5]. Definitive RT is a treatment option for advanced-stage, unresectable, medically inoperable tumors or for patients who refuse surgery [6].

Ultimately, around 70% of oral cavity cancers require RT as a form of definitive or postoperative radiation therapy, with or without chemotherapy. RT in oral cavity cancer can be delivered using various planning techniques, including two-dimensional radiotherapy (2D RT), three-dimensional conformal radiotherapy (3D-CRT), intensity-modulated radiotherapy (IMRT), and volumetric modulated arc therapy (VMAT). Advances in imaging, target-volume delineation, 3D reconstruction, and computer-optimized planning have shifted RT from conventional 2D techniques to 3D-CRT, enabling more precise beam shaping and better sparing of normal tissues. Further refinements in conformal RT led to the development of IMRT, in which simultaneous geometric and intensity modulation of radiation beams allows non-uniform fluence from multiple beam angles to optimize the composite dose distribution. These technological advances provide a dosimetric basis for comparing 3D-CRT and IMRT in terms of acute toxicity in locally advanced oral cavity cancer [7].

In Bangladesh, 3D-CRT is often used for oral cavity cancer RT because it is widely available and relatively cost-effective. However, advances in radiation technology - such as faster multileaf collimators (MLCs), treatment couches with greater degrees of freedom, improved beam optimization algorithms, faster dose calculation, and better imaging modalities (including integrated image-guided RT) - have made IMRT increasingly available. IMRT enables higher dose conformity to the primary tumor while reducing the dose to surrounding normal tissues compared with 3D-CRT. Oral cavity cancer is an ideal site for the IMRT technique because of the complex geometry of this area, which leads to more opportunities for radiation-related acute and late toxicities. Comparing treatment-related acute toxicities between 3D-CRT and IMRT in oral cavity cancer RT may provide future guidance for the treatment of such patients in Bangladesh.

## Materials and methods

Ethical consideration

This study was approved by the Institutional Review Board (IRB) of Bangladesh Medical University (BMU) (approval number: BSMMU/2022/12511). After IRB approval from BMU, permission was obtained from the Department of Clinical Oncology, BMU; Delta Hospital Limited; and Ahsania Mission Cancer and General Hospital in Dhaka, Bangladesh. The study was carried out in line with the Declaration of Helsinki and Good Clinical Practice guidelines. All patients were given an explanation of the study, including its risks and benefits. They were also informed that if any complication arose due to the intervention, they would receive treatment for that particular complication free of cost. It was also explained to them that they had the right to refuse or accept participation in the study. Before each patient's involvement in the study, signed informed consent was obtained. All data obtained during the study period from the patients were kept confidential.

Study location and setting

The study was conducted at BMU, Delta Hospital Limited, and Ahsania Mission Cancer and General Hospital in Dhaka, Bangladesh, from December 2022 to October 2023.

Selection criteria of patients

Criteria for Inclusion

The inclusion criteria were biopsy-proven squamous cell carcinoma in locally advanced and inoperable oral cavity cancer (LAIOCC); no prior RT; Eastern Cooperative Oncology Group (ECOG) performance status ≤2 [8]; leukocyte count >4,000/mm³, hemoglobin >10 g/dL, platelet count >150,000/mm³, serum bilirubin <1.5 mg/dL, serum transaminase <2.5 times the upper normal limit, serum creatinine <1.4 mg/dL, with corresponding eGFR ≥60 mL/min.

Criteria for Exclusion

The exclusion criteria were age below 18 years or above 73 years; distant metastasis; prior or concurrent malignancy; pregnancy or lactation; and other medical comorbidities for which patients were declared unfit for the proposed treatment modalities. This study was performed on 84 patients with LAIOCC.

Study design and treatment

This was a quasi-experimental study in which a total of 98 patients with LAIOCC were initially assessed for eligibility. Eight patients were excluded because they did not meet the selection criteria. Finally, following the application of the inclusion and exclusion criteria, 90 patients were equally divided between two arms (45 patients in Arm A and 45 patients in Arm B) using purposive sampling (Figure 1). Patients in both arms received CCRT to a total dose of 70 Gy in 35 fractions of 2 Gy each, with weekly cisplatin 40 mg/m²; Arm A was treated using the 3D-CRT technique, whereas Arm B was treated using IMRT. All patients were treated using an Elekta Versa HD® linear accelerator (manufactured by Elekta AB, Stockholm, Sweden) located at the Department of Clinical Oncology, BMU. In the 3D-CRT arm, static-field conformal plans were generated using forward planning with MLC shaping. In the IMRT arm, step-and-shoot inverse-planned treatments were delivered with daily image guidance using onboard kV imaging and/or cone-beam computed tomography (CBCT) to verify patient positioning before each fraction. Organs at risk (OARs) were contoured according to a single institutional protocol in both arms. Dose constraints were based on institutional protocols and applied consistently in both the 3D-CRT and IMRT arms. Key constraints included spinal cord Dmax < 45 Gy, brainstem Dmax < 54 Gy, mandibular Dmax < 70 Gy, and efforts to keep the mean dose to at least one parotid gland < 26 Gy while maintaining target coverage.

**Figure 1:**
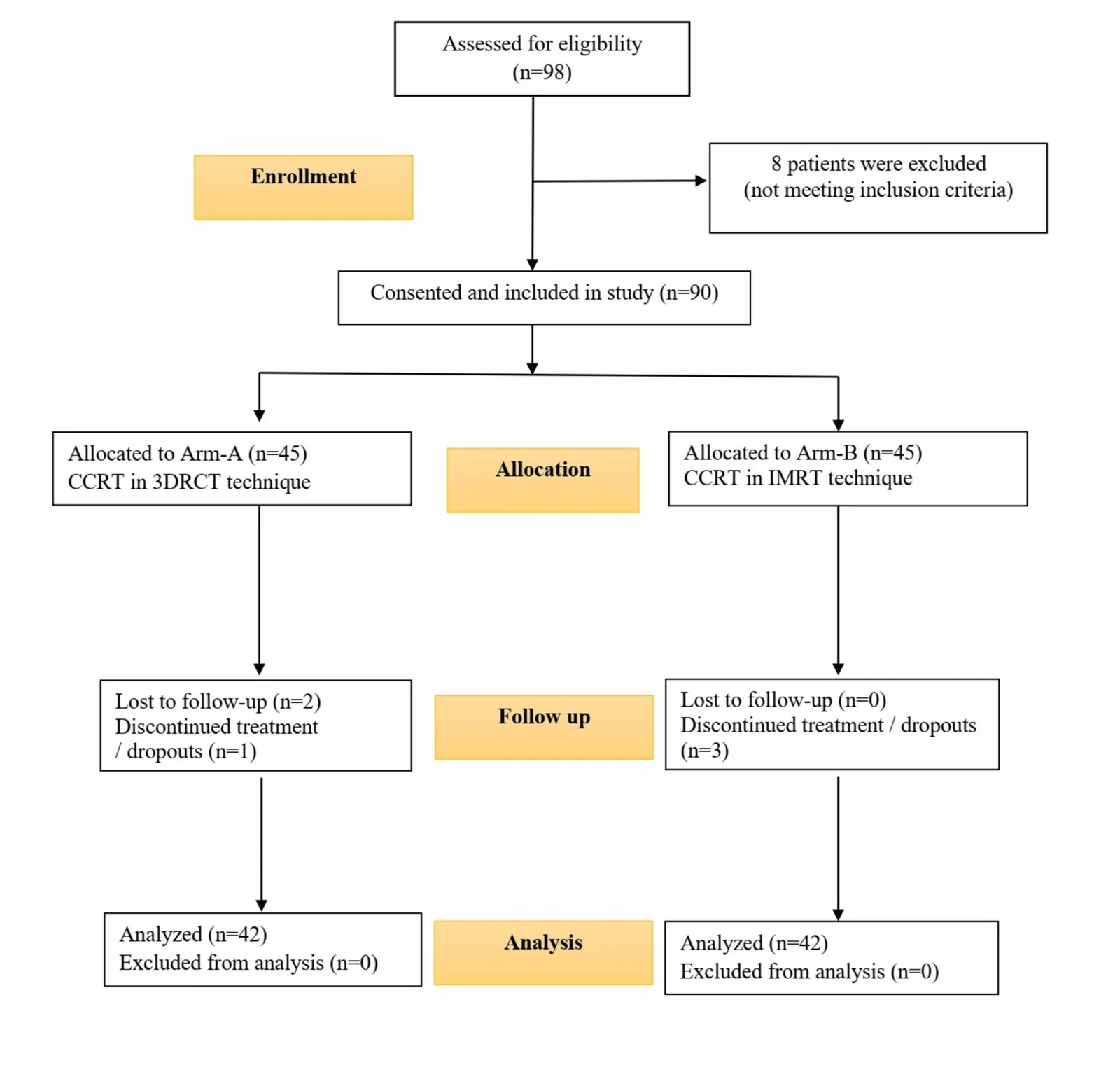
Flowchart showing the methods of patient enrollment, allocation, follow-up, and analysis

In cases of RT treatment interruption due to toxicities, a gap correction was made to the planned RT schedule, and the duration of the RT treatment interruption was recorded. During treatment, three patients in each arm discontinued treatment or were lost to follow-up (Figure 1). Ultimately, a total of 84 patients (42 patients in each arm) were included in the analysis of treatment-related toxicities. Chemotherapy completion, dose modification, and RT interruption were reported for these 84 patients.

Sample size calculation 

We needed 38 patients in each arm according to the following formula [9]: $n = \frac{p_1(1-p_1) + p_2(1-p_2)}{(p_1-p_2)^2} \times (Z_\alpha + Z_\beta)^2$, where p₁ = 0.85 and p₂ = 0.575 (in the 3D-CRT arm, dysphagia was expected to develop in 85% of cases, whereas in the IMRT arm, it was expected in 57.5% of cases [10]); Zα = 1.96 (from the Z table at a 5% level of significance with a 95% confidence interval); and Zβ = 0.84 (from the Z table at 80% power). With a 10% allowance for loss to follow-up, the final sample size was 42 patients in each arm.

Assessment and data collection

In this study, the outcome variables were acute toxicities. Patients in both arms were assessed weekly during CCRT, starting from the onset of RT and continuing until six weeks after completion of treatment. Acute non-hematological toxicities (dysphagia, xerostomia, mucositis, and dermatitis) and acute hematological toxicities (anemia, leukopenia, and thrombocytopenia) were assessed in each patient during this period. These toxicities were recorded and graded using the Common Terminology Criteria for Adverse Events (CTCAE), Version 5.0 [11], or the Toxicity Criteria of the Radiation Therapy Oncology Group (RTOG)/European Organization for Research and Treatment of Cancer (EORTC), 1995 [12]. To gather information, a data collection sheet was employed.

Statistical analysis

Data analysis was conducted according to the objectives of the study using IBM SPSS Statistics for Windows, Version 26 (Released 2018; IBM Corp., Armonk, NY, USA). In this study, quantitative variables (age) were compared using the t-test, and qualitative variables were compared using the chi-square test or Fisher's exact test. The test results were expressed in terms of p-values, and a p-value of less than 0.05 (<0.05) was considered statistically significant. Three patients in each arm did not complete the treatment or were lost to follow-up (Figure 1). Ultimately, 42 patients in each arm (total 84 patients) were evaluable for treatment-related toxicity analysis. Treatment completion, cisplatin-cycle delivery, dose modification, and RT interruption were summarized for these 84 patients.

## Results

In this study, the mean age of the 84 participants in both arms was 57.89 ± 6.91 years. In Arm A, the mean age was 58.95 ± 6.81 years, while in Arm B, the mean age was 56.83 ± 6.94 years. Overall, 65 (77.4%) patients were male and 19 (22.6%) patients were female. The male-to-female ratio was 3.42:1. Most of the patients (45.2%) in both arms were educated up to the primary level. The majority of patients in both arms were farmers, with 47.6% in Arm A and 50% in Arm B. Overall, 33.3% of patients in both arms had comorbidities such as diabetes mellitus, hypertension, and a previous history of ischemic heart disease. Most patients in this study had an ECOG performance status of 1 (64.3%) (Table 1).

**Table 1:** Characteristics of the patients and disease status Data are presented as mean ± standard deviation (SD) for continuous variables and as n (%) for categorical variables. ^a^ p-value calculated by an independent-sample t-test; ^b^ p-value calculated by Fisher’s exact test. p < 0.05 was considered statistically significant. ECOG: Eastern Cooperative Oncology Group; DM: Diabetes Mellitus; HTN: Hypertension; IHD: Ischemic Heart Disease; HSC: Higher Secondary Certificate

Characteristics	Total (n = 84)	Arm A (3D-CRT) (n = 42)	Arm B (IMRT) (n = 42)	t-value/χ² value	p-value
Age (mean± SD)	57.89 ± 6.91	58.95 ± 6.81	56.83 ± 6.94	1.41	0.16^a^
Gender					
Male	65 (77.4%)	33 (78.6%)	32 (76.2%)	0.07	1.00^b^
Female	19 (22.6%)	9(21.4%)	10 (23.8%)		
Educational status					
Upto Primary	38 (45.2%)	21 (50.0%)	17 (40.5%)	0.81	0.67^b^
Upto HSC	27 (32.1%)	12 (28.6%)	15 (35.7%)		
College and university	19 (22.6%)	9 (21.4%)	10 (23.8%)		
Occupation					
Farmer	41 (48.8%)	20 (47.6%)	21 (50.0%)	0.53	1.00^ b^
Service holder	11 (13.1%)	6 (14.3%)	5 (11.9%)		
Businessman	13 (15.5%)	7 (16.7%)	6 (14.3%)		
House wife	16 (19.1%)	8 (19.1%)	8 (19.1%)		
Others	3 (3.6%)	1 (2.8%)	2 (3.6%)		
Co-morbidities					
No	56 (66.7%)	24 (57.1%)	32 (76.2%)	3.93	0.41^ b^
DM	13 (15.5%)	9 (21.4%)	4 (9.5%)		
HTN	6 (7.1%)	4 (9.5%)	2 (4.8%)		
IHD	5 (6.0%)	3 (7.1%)	2 (4.8%)		
Others	4 (4.8%)	2 (4.8%)	2 (4.8%)		
ECOG performance status					
ECOG 0	13 (15.5%)	4 (9.5%)	9 (21.4%)	2.53	0.29^ b^
ECOG 1	54 (64.3%)	28 (66.7%)	26 (61.9%)		
ECOG 2	17 (20.2%)	10 (23.7%)	7 (16.7%)		
Primary site of tumor					
Anterior 2/3rd of tongue	10 (11.9%)	6 (14.3%)	4 (9.5%)	2.23	0.89^ b^
Buccal mucosa	21 (25.0%)	11 (26.2%)	10 (23.8%)		
Floor of the mouth	26 (31.0%)	12 (28.6%)	14 (33.3%)		
Alveolar ridge	21 (25.0%)	9 (21.4%)	12 (28.6%)		
Retromolar trigone	5 (6.0%)	3 (7.1%)	2 (4.8%)		
Hard palate	1 (1.2%)	1 (2.4%)	0 (0.0%)		
Stage of disease					
Stage III	27 (32.1%)	10 (23.8%)	17 (40.5%)	3.24	0.23^ b^
Stage IVA	38 (45.2%)	20 (47.6%)	18 (42.9%)		
Stage IVB	19 (22.6%)	12 (28.6%)	7 (16.7%)		
Histopathological grading					
Grade 1	19 (22.6%)	9 (21.4%)	10 (23.8%)	0.24	0.89^ b^
Grade 2	56 (66.7%)	29 (69.0%)	27 (64.3%)		
Grade 3	9 (10.7%)	4 (9.5%)	5 (11.9%)		

The most common risk factor observed among the patients was smoking, reported in 75% of patients in both arms. Other risk factors were betel nut chewing (57.1%), Jorda and tobacco leaf intake (50%), gul (23.8%), and alcohol consumption (4.8%) (Table 2).

**Table 2:** Distributions of risk factors and clinical features Data are presented as n (%). ^a^ p-value calculated by Fisher’s exact test. p < 0.05 was considered statistically significant. 3D-CRT, Three-Dimensional Conformal Radiotherapy; IMRT, Intensity-Modulated Radiation Therapy

Variables	Total (n = 84)	Arm A (3D-CRT) (n = 42)	Arm B (IMRT) (n = 42)	χ² value	p-value
Risk factors					
Smoking					
Yes	63 (75.0%)	31 (73.8%)	32 (76.2%)	0.06	1.00^ a^
No	21 (25.0%)	11 (26.2%)	10 (23.8%)		
Betel nut chewing					
Yes	49 (58.3%)	25 (59.5%)	24 (57.1%)	0.05	1.00^ a^
No	35 (41.7%)	17 (40.5%)	18 (42.9%)		
Jorda and tobacco leaf					
Yes	44 (52.4%)	23 (54.8%)	21 (50.0%)	0.19	0.83^ a^
No	40 (47.6%)	19 (45.2%)	21 (50.0%)		
Gul					
Yes	22 (26.2%)	12 (28.6%)	10 (23.8%)	0.25	0.81^ a^
No	62 (73.8%)	30 (71.4%)	32 (76.2%)		
Alcohol					
Yes	5 (6.0%)	3 (7.1%)	2 (4.8%)	0.21	1.00^ a^
No	79 (94.0%)	39 (92.9%)	40 (95.2%)		
Clinical features					
Pain in the mouth					
Yes	55 (65.5%)	26 (61.9%)	29 (69.0%)	0.47	0.65^ a^
No	29 (34.5%)	16 (38.1%)	13 (31.0%)		
Swelling in oral cavity					
Yes	60 (71.4%)	27 (64.3%)	33 (78.6%)	2.1	0.23^ a^
No	24 (28.6%)	15 (35.7%)	9 (21.4%)		
Ulceration					
Yes	44 (52.4%)	21 (50.0%)	23 (54.8%)	0.19	0.83^ a^
No	40 (47.6%)	21 (50.0%)	19 (45.2%)		
Neck swelling					
Yes	31 (36.9%)	15 (35.7%)	16 (38.1%)	0.05	1.00^ a^
No	53 (63.1%)	27 (64.3%)	36 (61.9%)		
Trismus					
Yes	18 (21.4%)	10 (23.8%)	8 (19.0%)	0.28	0.79^ a^
No	66 (78.6%)	32 (76.2%)	34 (81.0%)		

The most common site of oral cavity cancer in this study was the floor of the mouth (31%). Other common sites of involvement were the buccal mucosa (25%), alveolar ridge (25%), and anterior two-thirds of the tongue (11.9%). Most patients in both arms presented with stage IVA disease (45.2%). The remainder had stage III (32.1%) or stage IVB (22.6%) disease. The majority of oral cavity carcinomas in this study were moderately differentiated squamous cell carcinomas (66.7%) (Table 1).

Regarding clinical features, most patients presented with swelling (71.4%), pain (65.5%), and ulceration (52.4%) in the oral cavity. Other common clinical features were neck swelling (36.9%) and trismus (21.4%) (Table 2).

During the treatment period, patients in both arms were assessed weekly up to six weeks after completion of treatment for acute hematological and non-hematological toxicities. For each patient, the maximum CTCAE toxicity grade observed from the start of chemoradiotherapy through the end of the six-week post-treatment assessment was recorded. Xerostomia was significantly more frequent and severe in Arm A than in Arm B (grade 2 xerostomia in 76.2% vs. 52.4%; p = 0.04) (Table 3).

**Table 3:** Distributions of treatment related acute non-hematological toxicities Data are presented as n (%). ^a^ p-value calculated by Fisher’s exact test. p < 0.05 was considered statistically significant. 3D-CRT, three-dimensional conformal radiotherapy; IMRT, intensity-modulated radiation therapy

Toxicities	Total (n = 84)	Arm A (3D-CRT) (n = 42)	Arm B (IMRT) (n = 42)	χ² value	p-value
Xerostomia					
Grade 0	0 (0.0%)	0 (0.0%)	0 (0.0%)	5.18	0.04^a^
Grade 1	30 (35.7%)	10 (23.8%)	20 (47.6%)		
Grade 2	54 (64.3%)	32 (76.2%)	22 (52.4%)		
Grade 3	0 (0.0%)	0 (0.0%)	0 (0.0%)		
Grade 4	0 (0.0%)	0 (0.0%)	0 (0.0%)		
Dysphagia					
Grade 0	0 (0.0%)	0 (0.0%)	0 (0.0%)	6.98	0.02^a^
Grade 1	26 (31.0%)	8 (19.0%)	18 (42.9%)		
Grade 2	56 (66.7%)	32 (76.2%)	24 (57.1%)		
Grade 3	2 (2.4%)	2 (4.8%)	0 (0.0%)		
Grade 4	0 (0.0%)	0 (0.0%)	0 (0.0%)		
Mucositis					
Grade 0	0 (0.0%)	0 (0.0%)	0 (0.0%)	4.49	0.22^a^
Grade 1	16 (19.0%)	5 (11.9%)	11 (26.2%)		
Grade 2	47 (56.0%)	25 (59.5%)	22 (52.4%)		
Grade 3	19 (22.6%)	10 (23.8%)	9 (21.4%)		
Grade 4	2 (4.8%)	2 (2.4%)	0 (0.0%)		
Dermatitis					
Grade 0	0 (0.0%)	0 (0.0%)	0 (0.0%)	1.23	0.63^a^
Grade 1	18 (21.4%)	11 (26.2%)	7 (16.7%)		
Grade 2	61 (72.6%)	29 (69.0%)	32 (76.2%)		
Grade 3	5 (6.0%)	2 (4.8%)	3 (7.1%)		
Grade 4	0 (0.0%)	0 (0.0%)	0 (0.0%)		

Dysphagia was also significantly more common in Arm A, with higher proportions of grade 2 and grade 3 events (76.2% and 4.8% vs. 57.1% and 0%, respectively; p = 0.02) (Table 3). In contrast, the distribution of oral mucositis and radiation dermatitis grades did not differ significantly between the arms (mucositis, p = 0.22; dermatitis, p = 0.63), although all patients experienced at least grade 1 mucositis and dermatitis (Table 3).

Acute hematological toxicities were also similar between the two arms. Grade 1 anemia occurred in 73.8% and grade 2 anemia in 8.3% of patients overall; grade 1 leukopenia and thrombocytopenia were observed in 20.2% and 13.1%, respectively. No grade 3 or 4 hematological toxicities were recorded, and none of these differences reached statistical significance (Table 4).

**Table 4:** Distributions of treatment-related hematological toxicities Data are presented as n (%). ^a^ p-value calculated by Fisher’s exact test. p < 0.05 was considered statistically significant. 3D-CRT, three-dimensional conformal radiotherapy; IMRT, intensity-modulated radiation therapy

Toxicities	Total (n=84)	Arm A (3D-CRT) (n=42)	Arm B (IMRT) (n=42)	χ² value	p-value
Anemia					
Grade 0	15 (17.9%)	9 (21.4%)	6 (14.3%)	0.81	0.75^a^
Grade 1	62 (73.8%)	30 (71.4%)	32 (76.2%)		
Grade 2	7 (8.3%)	3 (7.1%)	4 (9.5%)		
Grade 3	0 (0.0%)	0 (0.0%)	0 (0.0%)		
Grade 4	0 (0.0%)	0 (0.0%)	0 (0.0%)		
Leukopenia					
Grade 0	67 (79.8%)	33 (78.6%)	34 (81.0%)	0.07	1.00^a^
Grade 1	17 (20.2%)	9 (21.4%)	8 (19.0%)		
Grade 2	0 (0.0%)	0 (0.0%)	0 (0.0%)		
Grade 3	0 (0.0%)	0 (0.0%)	0 (0.0%)		
Grade 4	0 (0.0%)	0 (0.0%)	0 (0.0%)		
Thrombocytopenia					
Grade 0	73 (86.9%)	35 (83.3%)	38 (90.5%)	0.94	0.52^a^
Grade 1	11 (13.1%)	7 (16.7%)	4 (9.5%)		
Grade 2	0 (0.0%)	0 (0.0%)	0 (0.0%)		
Grade 3	0 (0.0%)	0 (0.0%)	0 (0.0%)		
Grade 4	0 (0.0%)	0 (0.0%)	0 (0.0%)		

In this study, 85.7% of patients completed all seven planned cisplatin cycles. Eight patients (9.5%) completed six cycles, whereas four patients (4.8%) completed five cycles. The distribution of cisplatin cycles completed did not differ significantly between the treatment arms (p = 0.71). Dose modification was required in five patients (6.0%) (p = 1.00) (Table 5).

**Table 5:** Cisplatin cycle completion, dose modification, and radiotherapy interruption Data are presented as n (%). ^a^ p-value calculated by Fisher’s exact test. p < 0.05 was considered statistically significant. 3D-CRT, three-dimensional conformal radiotherapy; IMRT, intensity-modulated radiation therapy

Variables	Total (n = 84)	Arm A (3D-CRT) (n = 42)	Arm B (IMRT) (n = 42)	χ² value	p-value
Cisplatin cycles completed					
7 cycles	72 (85.7%)	35 (83.3%)	37 (88.1%)	1.05	0.71^a^
6 cycles	8 (9.5%)	4 (9.5%)	4 (9.5%)		
5 cycles	4 (4.8%)	3 (7.1%)	1 (2.4%)		
Cisplatin dose modification					
Yes	5 (6.0%)	3 (7.1%)	2 (4.8%)	0.21	1.00^ a^
No	79 (94.0%)	39 (92.9%)	40 (95.2%)		
Radiotherapy interruption					
No	62 (73.8%)	30 (71.4%)	32 (76.2%)	4.28	0.12^ a^
≤ 1 week	18 (21.4%)	8 (19.0%)	10 (23.8%)		
> 1 week	4 (4.8%)	4 (9.5%)	0 (0.0%)		

RT interruptions occurred in 26.2% of patients. Eighteen patients (21.4%) experienced an interruption of up to one week, including eight (19.0%) in the 3D-CRT arm and ten (23.8%) in the IMRT arm. Four patients (4.8%) experienced an interruption exceeding one week; all four were in the 3D-CRT arm. The difference between the groups was not statistically significant (p = 0.12) (Table 5). RT gap correction was applied after treatment interruptions according to the institutional protocol.

## Discussion

In this study, the mean age of the participants was 57.89 ± 6.91 years, which is consistent with regional data showing that HNCs predominantly present in the sixth and seventh decades of life. Overall, 77.4% of patients were male and 22.6% were female (male-to-female ratio, 3.42:1), a pattern similar to that reported in South Asian patients, where oral cavity cancer incidence in men is more than twice that in women [13]. Smoking was reported in 76.2% of patients. Other common risk factors were betel nut chewing, Jorda and tobacco leaf intake, gul, and alcohol consumption. Involvement of the floor of the mouth was observed in 31% of patients, followed by the buccal mucosa (25%), alveolar ridge (25%), and anterior two-thirds of the tongue (11.9%). Bhattacharjee et al. (2021) also found a similar pattern of risk factor distribution among oral cavity cancer patients in West Bengal, India, with buccal mucosa (34%) being the most common site of oral cavity cancer [14]. Although the oral tongue is the more common subsite in the USA and European countries, buccal mucosa, gingiva, and floor of the mouth are more common sites of oral cavity cancer in South Asian countries [15], because betel nut and tobacco leaf chewing are more common risk factors in this region [16]. 

Both 3D-CRT and IMRT techniques are widely accepted methods of radiation treatment delivery in patients with locally advanced, inoperable oral cavity cancer. Irradiation of HNC can cause damage to the parotid and submandibular glands. This damage causes hypofunction of the salivary glands, resulting in xerostomia. In this study, xerostomia was more frequently observed in the 3D-CRT arm. Regarding xerostomia, only grade 1 and grade 2 toxicities were observed, and no patients developed grade 3 toxicities. Grade 2 xerostomia was significantly more frequent in Arm A (3D-CRT) than in Arm B (IMRT) (76.2% vs. 52.4%; p = 0.04). Ghosh et al. (2016) also observed a statistically significant difference in grade 2 xerostomia (p = 0.023), with 72.5% of patients in the 3D-CRT group and 45% in the IMRT group experiencing this toxicity during HNC RT, which correlates with the findings of this study [10]. Reddy et al. (2022) also found that, compared with 3D-CRT, IMRT resulted in lower rates of xerostomia in patients with oral cancers based on dosimetric and subjective analyses [17].

Dysphagia was also observed more frequently in the 3D-CRT arm than in the IMRT arm. In Arm A, 8 (19%), 32 (76.2%), and 2 (2.4%) patients developed grade 1, grade 2, and grade 3 dysphagia, respectively. In Arm B, 18 (42.9%) and 24 (57.1%) patients developed grade 1 and grade 2 dysphagia, respectively. This difference was statistically significant (p = 0.02). Ghosh et al. (2016) also found a statistically significant difference in dysphagia between the 3D-CRT group (85% of total cases) and the IMRT group (45% of total cases), which correlates with the findings of this study [10]. Kucha et al. (2020) also observed more grade 2 and grade 3 dysphagia in the 3D-CRT arm compared with the IMRT arm (p = 0.029) in patients with locally advanced HNC [18]. 

All patients in both arms developed oral mucositis (grade 1: 19.0%, grade 2: 56.0%, grade 3: 22.6%, grade 4: 4.8%) and radiation dermatitis (grade 1: 21.4%, grade 2: 72.6%, grade 3: 6.0%), with no significant difference between the 3D-CRT and IMRT arms. Gupta et al. (2012) also mentioned the development of mucositis and dermatitis in all patients receiving RT with either the 3D-CRT or IMRT technique for head and neck squamous cell carcinoma [19]. In our study, the distribution of mucositis was not significantly different (p = 0.22) between the two arms, which correlates with the findings of Ghosh et al. (2016) [10]. The distribution of radiation-induced dermatitis was also not significantly different (p = 0.63), which correlates with the findings of Gupta et al. (2012) [19]. Hematological toxicities (anemia, leukopenia, and thrombocytopenia) were comparable between the two arms, with no statistically significant differences.

Cisplatin cycle number, dose modification, and RT treatment interruption were broadly comparable between the two arms. Most patients completed all seven planned weekly cisplatin cycles, with completion rates of 83.3% in the 3D-CRT arm and 88.1% in the IMRT arm. Cisplatin dose modification was uncommon and did not differ significantly between the treatment groups. RT interruption occurred in 22 of 84 patients (26.2%). Although all four interruptions lasting more than one week occurred in the 3D-CRT arm, RT interruptions did not differ significantly between the arms.

This study has several limitations. First, it was conducted over a one-year period; therefore, overall survival and late toxicities could not be assessed, and only acute toxicities during treatment and up to six weeks post-therapy were evaluated. Second, the study was unblinded, non-randomized, and quasi-experimental, so selection bias cannot be excluded.

## Conclusions

In this study comparing CCRT delivered by 3D-CRT versus IMRT in patients with LAIOCC, the IMRT technique was associated with a significantly lower incidence of acute xerostomia and dysphagia. Other acute toxicities, including oral mucositis, radiation dermatitis, and hematological events (anemia, leukopenia, and thrombocytopenia), occurred at comparable rates in both treatment arms.

These findings indicate that IMRT provides a clinically meaningful advantage in reducing salivary gland- and swallowing-related side effects during and immediately after treatment. When the technology is available, IMRT should be preferred for this group of patients to improve treatment tolerability.
